# Variation in habitat soundscape characteristics influences settlement of a reef-building coral

**DOI:** 10.7717/peerj.2557

**Published:** 2016-10-13

**Authors:** Ashlee Lillis, DelWayne Bohnenstiehl, Jason W. Peters, David Eggleston

**Affiliations:** 1 Woods Hole Oceanographic Institution, Woods Hole, MA, United States; 2 Department of Marine, Earth, and Atmospheric Sciences, North Carolina State University, Raleigh, NC, United States; 3 Artificial Reef Program, North Carolina Division of Marine Fisheries, Morehead City, NC, United States of America

**Keywords:** Coral, Larval settlement, Reef soundscape, Habitat cue, Habitat selection

## Abstract

Coral populations, and the productive reef ecosystems they support, rely on successful recruitment of reef-building species, beginning with settlement of dispersing larvae into habitat favourable to survival. Many substrate cues have been identified as contributors to coral larval habitat selection; however, the potential for ambient acoustic cues to influence coral settlement responses is unknown. Using *in situ* settlement chambers that excluded other habitat cues, larval settlement of a dominant Caribbean reef-building coral, *Orbicella faveolata*, was compared in response to three local soundscapes, with differing acoustic and habitat properties. Differences between reef sites in the number of larvae settled in chambers isolating acoustic cues corresponded to differences in sound levels and reef characteristics, with sounds at the loudest reef generating significantly higher settlement during trials compared to the quietest site (a 29.5 % increase). These results suggest that soundscapes could be an important influence on coral settlement patterns and that acoustic cues associated with reef habitat may be related to larval settlement. This study reports an effect of soundscape variation on larval settlement for a key coral species, and adds to the growing evidence that soundscapes affect marine ecosystems by influencing early life history processes of foundational species.

## Introduction

Coral reefs are one of the most diverse and productive ecosystems on Earth, supporting 25% of all marine fish in just 0.1% of ocean area, and providing far-reaching ecological and economic benefits (Moberg & Folke, 1999). The scleractinian corals that create the biogenic physical structure of reef habitat are fundamental to the existence of coral reef communities, and live coral abundance drives the ecological processes of a plethora of reef-dependent fish and invertebrates (Ritson-Williams et al., 2009). Reef-building corals, like the majority of benthic organisms, produce planktonic larvae that disperse via ocean currents for hours to weeks before settling and metamorphosing to become site-attached adults (Baird, Guest & Willis, 2009). Larval settlement and habitat selection are key determinants of coral recruitment, survival, and reproductive success (Babcock & Mundy, 1996; Baird, Babcock & Mundy, 2003; Harrington et al., 2004), and an influx of larvae is critical to maintaining and recovering coral populations (Gleason & Hofmann, 2011). Despite their importance, early life history processes remain least understood of all life stages, and how variation in environmental factors influences the transition from pelagic to benthic existence for these organisms is not well known. Until relatively recently, dispersing larvae were considered passive particles, yet contemporary evidence demonstrates that even weakly swimming invertebrate larvae such as coral planulae exert considerable control over their dispersal via vertical movement in the water column, and that settling larvae select or reject attachment sites in response to a suite of physical, chemical and biological factors over multiple scales (Kingsford et al., 2002; Gleason & Hofmann, 2011).

Settlement cues may be pivotal both in facilitating larval encounter with reef substrate, and in determining the specific locations in which coral planulae attach. Numerous water-borne chemical compounds produced by algae and microbes have been implicated in the recruitment patterns of coral by inducing or inhibiting settlement (Kuffner et al., 2006; Vermeij et al., 2008; Dixson, Abrego & Hay, 2014), and larvae are known to respond to light levels (Mundy & Babcock, 1998), as well as physical and biological substrate properties (e.g., texture, colour, crustose coralline algae, biofilms) (Heyward & Negri, 1999; Mason, Beard & Miller, 2011). Most recently, the soundscape (i.e., the combination of physical and biological sounds in a particular location) has garnered attention as an additional sensory cue for larvae (Montgomery et al., 2006; Simpson et al., 2008; Stanley, Radford & Jeffs, 2012). The acoustic characteristics of the marine environment have the potential to provide rich sensory information to settling organisms, reflecting both the presence and quality of the adult habitat over relatively broader spatial scales (e.g., meters to kilometers) than localized chemical and substrate cues (Radford, Stanley & Jeffs, 2014; Lillis, Eggleston & Bohnenstiehl, 2014; Piercy et al., 2014). Acoustic characteristics have been implicated in the orientation and settlement of larval fishes, crustaceans, and molluscs (Simpson et al., 2004; Stanley, Radford & Jeffs, 2012; Lillis, Eggleston & Bohnenstiehl, 2013; Lillis, Bohnenstiehl & Eggleston, 2015), and a variety of marine invertebrates are known to be sensitive to the water- and substrate-borne vibrations (i.e., particle motions) generated by sound waves (Budelmann, 1989; Budelmann, 1992).

Cnidarian sensory hair bundles are very similar to vertebrate auditory hair cells (Arkett, MacKie & Meech, 1988; Watson, Mire & Hudson, 1997), and because coral planula larvae are densely covered with sensory hairs it is hypothesized that they may be sensitive to sound vibrations (Vermeij et al., 2010). Like other marine invertebrate larvae lacking a specialized gas-filled auditory organ, cnidarian larvae would likely be sensitive to the particle motion component of acoustic stimuli rather than capable of detecting pressure changes associated with sound waves (Budelmann, 1992; Mooney et al., 2010). A single replay experiment showed that cultured larvae of the dominant Caribbean reef building coral *O. faveolata* moved toward a combination of sounds from coral reefs in a contained low flow environment (Vermeij et al., 2010); however, this finding has not been investigated further, and the significance of habitat-related acoustic cues to coral settlement patterns remains unknown. To examine the influence of ambient reef soundscape variation on coral larval settlement, the settlement response of *O. faveolata* larvae in chambers deployed at reefs that differed in habitat quality and acoustic characteristics was compared in this study.

## Methods

### Gamete collection and larval rearing

**Figure 1:**
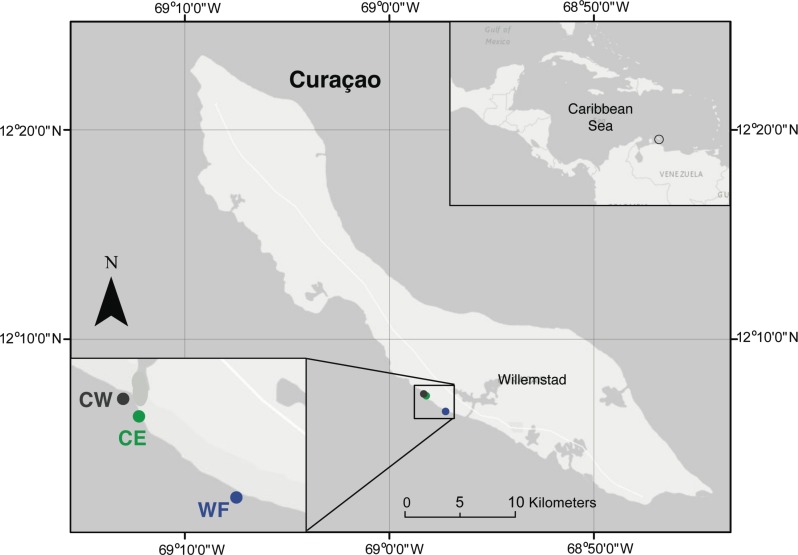
Map of the island of Curaçao. Locations of experimental reef sites: Water Factory (WF), Carmabi East (CE), and Carmabi West (CW). Coral gametes were collected at the Water Factory reef site.

Larvae of *O. faveolata*, commonly known as mountainous star coral, were laboratory reared following their annual mass spawning on 16 September 2014 in the Caribbean island of Curaçao. Gamete bundles from eight parent colonies were collected by tenting colonies during spawning events, at depths of 5–8 meters from a reef site known as “Water Factory” (12°6^′^34^′′^ N, 68°57^′^23^′′^ W; Fig. 1, WF). All field collections and experiments were conducted under the permissions and collecting permit 48,584 granted to CARMABI by the Government of Curaçao (Ministry of Health, Environment, and Nature).

Following nighttime collection, coral gametes were pooled and transported to the CARMABI research station laboratory, where they were allowed to fertilize for 120 min. Embryos were transferred to 1-L polystyrene containers using 0.45µm-filtered seawater, held at ambient temperature (28–29 °C), and cultured to swimming planula stage larvae with frequent monitoring. Culture water was changed as necessary to keep cultures clean and remove dead larvae (following methods detailed in: Vermeij et al., 2008). Seawater used in larval cultures and the experimental chambers described below was obtained by filtering the ambient CARMABI seawater using a 0.45 µm Millipore™ membrane filter. Larval cultures were closely monitored for the onset of settlement competence, and experimental trials started when larvae first began to exhibit settlement behaviours (i.e., bottom-seeking and searching along surfaces), which occurred ∼ 4 days following spawning. Only actively swimming larvae near the surface of culture containers were selected for experimental use since bottom-crawling larvae were considered to have reached advanced stages in the settlement process, and our goal was to present potential acoustic stimuli to larvae early in their competency period.

### Study sites

Three reef sites in proximity to the CARMABI marine biological station were selected as experimental locations (Fig. 1: Water Factory, WF; Carmabi East, CE; Carmabi West, CW). Sites were chosen to be in close proximity to each other, yet encompass a range in reef characteristics based on previous surveys of Curacao reef health by CARMABI scientists (Vermeij, 2012). The sites represented clearly visible qualitative differences in coral cover/reef condition, and preliminary sound recordings showed differences in acoustic characteristics (e.g., sound pressure levels and frequency content) between the sites. CW reef is located on the northwest side of the channel leading to Piscadera Baai (adjacent to CARMABI station), and primarily consists of sponge and coral rubble with little live coral or three-dimensional structure. CE reef is southeast of the Piscadera Baai channel, with a higher amount of live coral colonies, sponge cover and reef fish than CW, but present also are substantial industrial debris and algal growth on coral rubble. WF reef is a nearby site (<2 km) where live coral is abundant and diverse, fish are numerous, and is the location where coral spawning collections were conducted. All three sites also had similar frequent daytime exposure to small diving and fishing boats, as well as larger vessels commonly moored offshore around the island. An important consideration in selecting sites within a small area (less than 2 km) was avoidance of variability in other environmental factors that could influence coral settlement, such as water temperature. The area in which sites were located is known to have consistent current patterns and previous temperature measurements at WF and CARMABI reefs show highly correlated water temperatures with non-significant differences between sites in the mean temperature (M Vermeij, 1998, unpublished data). Moreover, the differences between the water temperatures at the sites are typically less than the accuracy of temperature loggers (0.2 °C), and less than differences reported not to influence coral larval settlement rates (>2 °C Nozawa & Harrison, 2007; Heyward & Negri, 2010).

### Settlement experiments

To determine if the soundscape at different reef sites influenced coral larval settlement, three replicate groups of 100 *O. faveolata* larvae were randomly selected and placed in watertight 80 mL polyethylene settlement chambers, and deployed at each of three field reef sites (9 chambers total in each trial). Larval settlement chambers at each reef site were positioned 0.5 m above the seabed in small patches of sand, and were positioned at least 5 m from each other in 8 m water depth. Chambers were securely attached to the bottom using metal stakes so that they did not move with water flow. Larvae were completely contained in the polyethylene chambers for the duration of experiments, allowing exposure to the ambient soundscapes while excluding any chemical cues associated with the reefs. Attenuation of sound pressure levels (frequency band: 50–20,000 Hz) across the thin polyethylene material were determined to be <2 dB in lab measurements. Chamber lids were opaque, with frosted semi-transparent sides so that larvae were exposed to the ambient day-night cycles. Chambers were filled with 0.45 µm-filtered seawater, and contained a ceramic settlement tile (3.4-cm diameter three-prong pottery stilt) at the bottom that had been conditioned in raw flowing seawater for one-month (under ambient temperature and light conditions) to establish crustose coralline algae on its surface (the preferred settlement substrate for coral larvae) as a realistic attachment surface for the study animals. Tiles were randomly assigned to the experimental chambers, and provided sufficient settlement surface for all larvae in each culture, i.e., substrate was not limiting. The settlement tiles were rinsed with 0.45 µm-filtered seawater and lightly scrubbed to remove non-encrusting organisms that may have attached during the conditioning period.

The experiment consisted of two *in situ* larval settlement trials conducted 21–25 September 2014 (resulting in six replicates total per treatment), the first trial lasting 48-hours and the second 24-hours. Trial length was determined based on concomitant larval settlement observed in lab larval cultures, with the aim of attaining sufficient settlement to measure possible treatment effects without being so long as to have complete settlement in any culture. At the conclusion of each trial, replicate larval chambers were collected from sites and transported to the laboratory where the number of settled larvae on tile surfaces was counted under a dissecting microscope. For this experiment, settlement was defined as securely attached or metamorphosed planulae at the conclusion of a trial. No evidence of larval mortality was visible in experimental chambers following the short trials or in lab cultures over the same period. Statistical analysis was implemented in MATLAB using an ANOVA procedure to test for significant differences in the numbers of settled larvae in settlement chambers deployed at the three reef sites, including site and trial as factors. No significant interaction was found between site and trial, thus this interaction was excluded from the analysis, and trial was treated as a blocking factor. A post-hoc Tukey’s HSD test was applied to evaluate significance of pair-wise comparisons among mean settlement (measured as settlers/culture).

### Acoustic monitoring

To compare the soundscape characteristics of the three reef sites during the experiment, a self-contained acoustic recorder (SoundTrap 201, Ocean Instruments, flat frequency response: 20 Hz–60 kHz) was deployed at each site, 0.5 m above the seabed and in the middle of the three replicate larval settlement chambers. Recorders simultaneously sampled at each site for 2-minutes every 10-minutes at a 96 kHz sampling rate, for the duration of the experimental period. Following retrieval, digital recording samples were analyzed using purpose-written MATLAB code to quantify and compare the ambient soundscapes to which larvae had been exposed during trials.

To examine differences in the overall spectral composition of the soundscapes at each site, a median acoustic spectrum (sound power as a function of frequency) was generated from the recordings at each site within a lower (25–1,000 Hz) and higher (3,000–20,000 Hz) frequency bandwidth known to have distinct sound sources. The lower frequency range includes most coral reef fish vocalizations, and is also influenced by wind-generated noise and vessel noise (Wenz, 1962; Lobel, Kaatz & Rice, 2010). The higher frequency band is dominated by the broadband crackling sounds produced by closure of the snapping claw of *Alpheus* and *Synalpheus* spp. shrimp (Johnson, Everest & Young, 1947). The spectrum between the two analysis bands includes frequencies where fish calls and snaps overlap significantly, making it difficult to disentangle their contributions to sound pressure levels. Following inspection of spectra, the root-mean-square sound pressure level (SPL) was calculated in each 2-min recording to compute median SPL at each site in the two bands of interest. To further characterize differences between the experimental site soundscapes that might reflect differences in reef properties, snapping shrimp snaps were counted using an envelope correlation method to generate a snap rate in detections per minute (Bohnenstiehl, Lillis & Eggleston, 2016). Because snapping shrimp are closely associated with habitat structure, and can relate to reef characteristics such as coral cover, snap count is often used as an acoustic metric for site comparisons (Radford et al., 2010).

To test if the median SPLs in each bandwidth and snap counts differed among reef sites, non-parametric Kruskal-Wallis analyses of variance were conducted, followed by Tukey-Kramer multiple comparison tests to determine which sites differed significantly in each acoustic variable.

## Results

**Figure 2:**
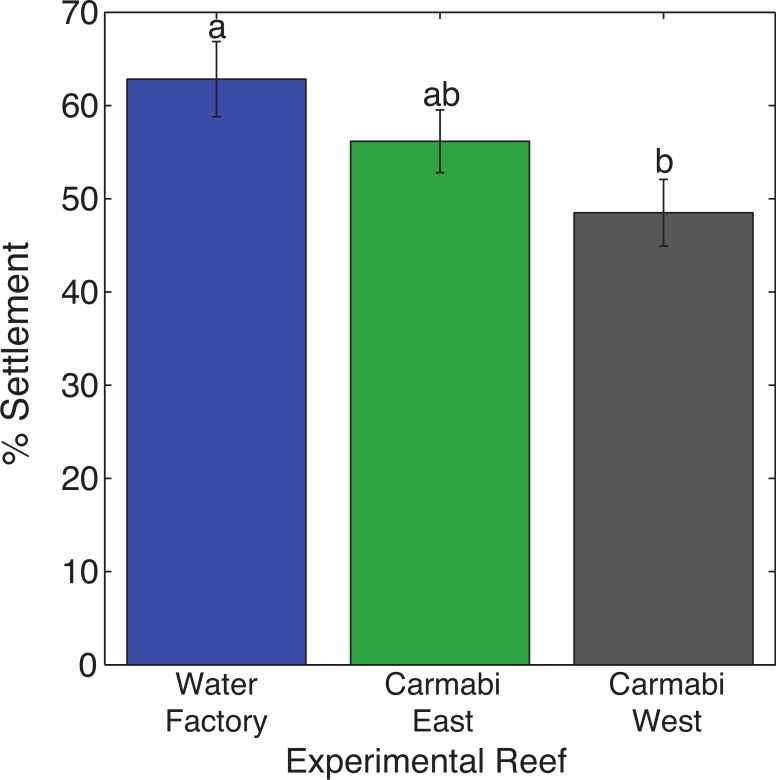
Coral settlement in response to soundscape variation. Settlement of *Orbicella faveolata* planulae (mean % settled per chamber ± 1 S.E.) in cultures deployed at each experimental reef site (*n* = 6, pooled results from two trials). Letters above bars indicate significant differences (*p* < 0.01) between values based on Tukey’s HSD test.

Reef site had a significant effect on larval settlement (F_2,14_ = 7.84, *p* < .01), with 1.3 times higher numbers of settled larvae (62.8% vs. 48.5% mean settlement rate) at the termination of trials for larval cultures deployed at WF reef compared to those at the CW reef (Fig. 2). Larval settlement values for cultures at the CE reef overlapped with the other two sites.

**Figure 3:**
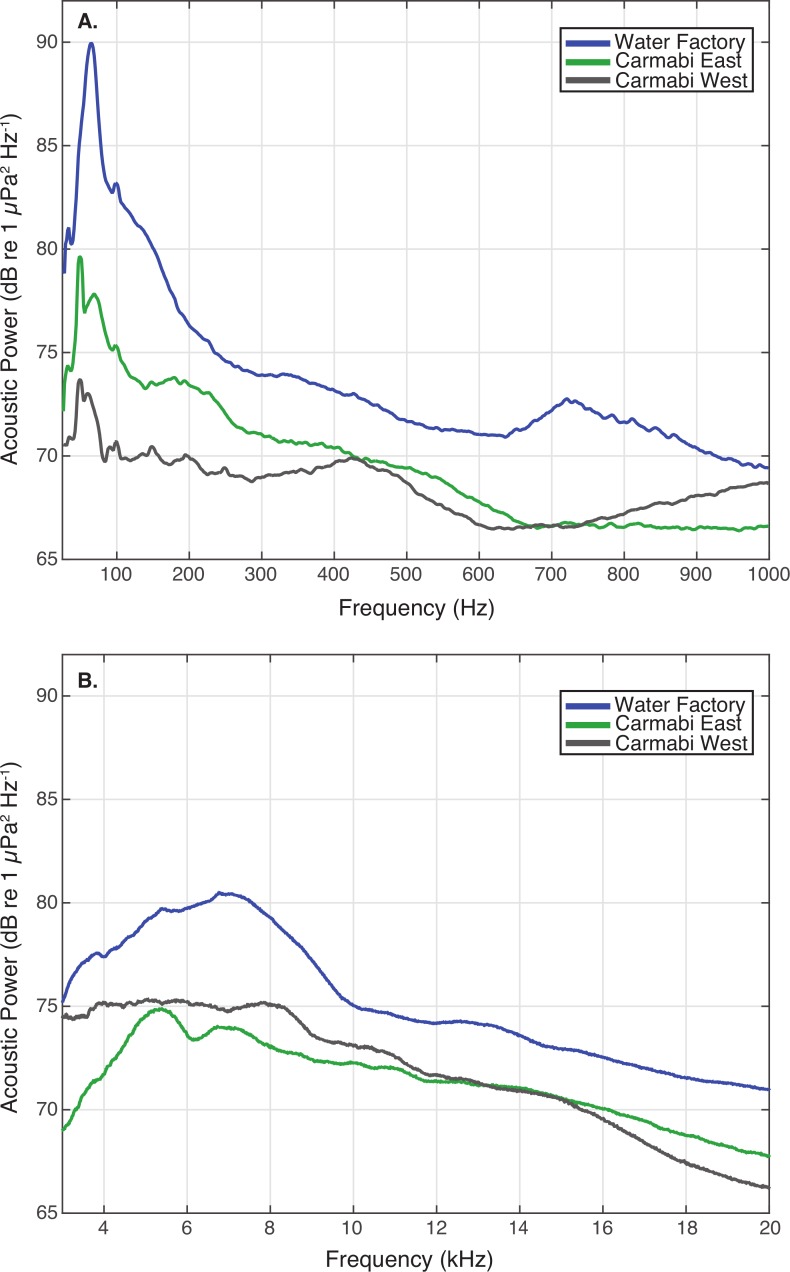
Reef site acoustic spectra. Median acoustic spectra for recordings collected at each experimental site during the settlement experiment, in (A) a lower frequency bandwidth (25–1,000 Hz), and (B) upper frequency bandwidth (3–20 kHz). The median power spectrum was determined for each site using spectra generated for 2-minute samples taken every 10-minutes throughout the settlement trials (September 21–25), using 0.5-second non-overlapping Hanning windows.

**Figure 4:**
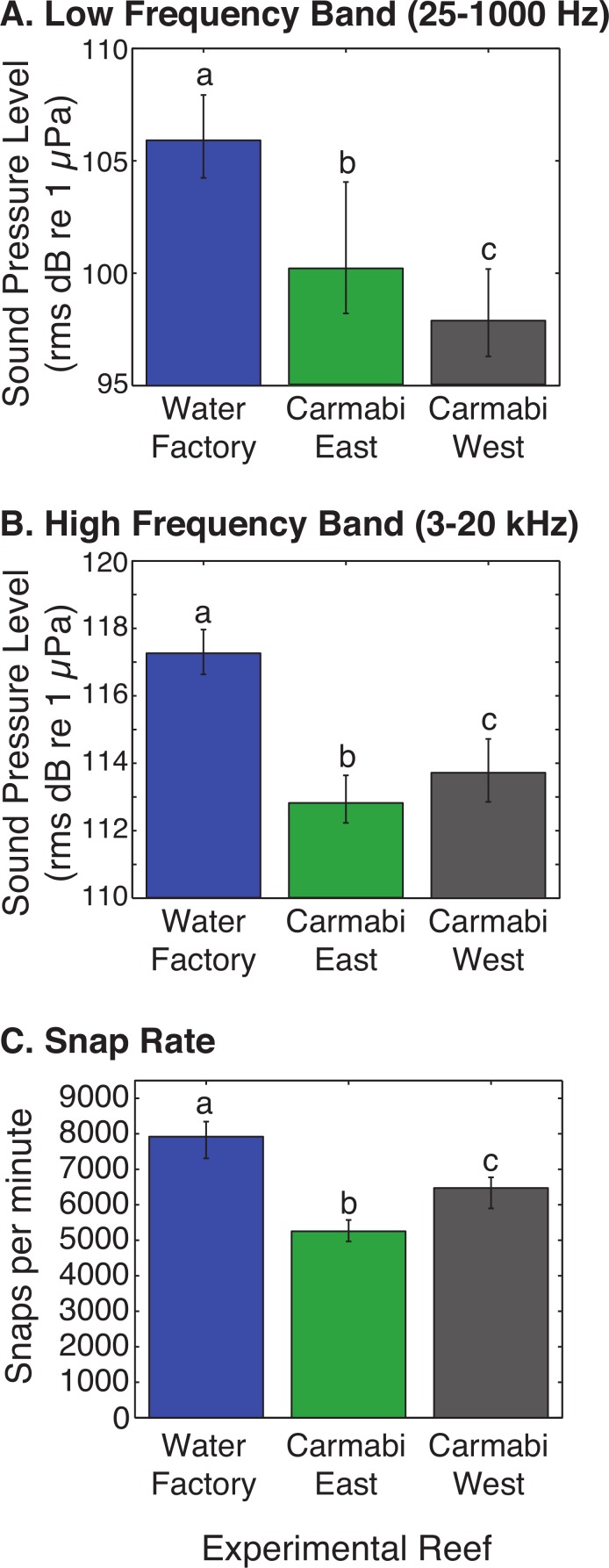
Comparison of reef site soundscape characteristics. Experimental reef site root-mean-square sound pressure levels (median) calculated for (A) low frequency band (25–1,000 Hz) and (B) high frequency band (3,000–20,000 Hz). Snap rate (median snaps per minute) at each reef site is shown in (C). Error bars represent the interquartile range and different letters denote statistically significant differences.

WF reef showed the highest overall acoustic power across both lower and upper frequencies compared to the other two sites (Fig. 3A and 3B), while the CW site soundscape produced the lowest overall acoustic power levels. CW recordings also produced a different spectral shape compared to the WF and CE sites, with a flatter spectrum observed from 100–400 Hz and increasing sound levels from 800–1,000 Hz. The WF spectrum shows large peaks at 60–70 Hz and 650–800 Hz that are absent from the two lower quality reef sites (Fig. 3A). In the upper frequency range, the CW spectrum shows lower sound levels from 3–12 kHz, where invertebrate-produced sounds dominate reef soundscapes. Median sound pressure levels in both lower and higher frequency bands were significantly different between sites, decreasing from WF to CE to CW in the lower frequencies (Fig. 4A). In the higher frequency band, median sound levels at WF were significantly higher, but CW had higher levels compared to CE across this range (Fig. 4B). This soundscape difference between the sites was also reflected by snap rate data (Fig. 4C).

## Discussion

This study reveals a settlement response by coral larvae to variability in ambient reef soundcape. By directly using the ambient reef soundscapes as acoustic treatments, and selecting reefs that differ in habitat characteristics, the results demonstrate that existing variation in the acoustic environment of different reef environments can influence the attachment of a reef-building coral. The settlement results corresponded to overall differences in sound levels measured between the sites during the experiment. The elevated larval settlement in chambers deployed at the loudest and most high quality reef site suggests that larvae may use reef sound quantity and/or quality as part of their habitat selection at settlement, and that a higher number of larvae may settle under certain acoustic conditions within a given timeframe even when presented with ample suitable substrate. This implies that reef soundscape cues could be important to coral recruitment patterns, and that habitat alterations that affect acoustic characteristics could affect recolonization of these habitats. If degraded reefs lack the appropriate acoustic cues to induce settlement, or produce acoustic cues that deter settlement, reef restoration and recovery could be inhibited, and habitat degradation perpetuated. This type of reinforcing mechanism is thought to occur for reef-associated chemical settlement factors (Lecchini et al., 2014; Dixson, Abrego & Hay, 2014). Increasing knowledge of acoustically-mediated settlement responses of coral larvae could be similarly important to understanding patterns of reef health and recovery after disturbance.

Our results show that settling coral larvae have the potential to incorporate soundscape cues into their habitat selection process, but the relative importance and scales of acoustic cues versus other habitat cues has yet to be determined. As other authors note, the coral settlement process is complex and consists of responses to a hierarchy of cues over different scales and in combination (Mason, Beard & Miller, 2011; Gleason & Hofmann, 2011). *Orbicella* spp. have been reported to select settlement sites based on the presence of crustose coralline algae (CCA) species (Ritson-Williams et al., 2013; Ritson-Williams, Arnold & Paul, 2016), biofilm-produced chemical cues (Sneed et al., 2014), as well as fine-scale differences in crustose coralline algae (CCA) distribution on substrates and orientation of substrate surfaces (Szmant & Miller, 2006). These responses are likely important in determining coral habitat selection upon larval contact with the reef, and much of the coral settlement literature has focused on testing these chemical/physical cues, but the current study further adds acoustic characteristics as component of the habitat selection cuescape. Effect sizes measured in these types of settlement assays, using closed cultures of larvae, are difficult to extrapolate to assess the ecological relevance of a particular cue to field populations or to adequately compare the importance of various cues measured in different studies. Because the larvae in these experiments were contained and provided ample attractive substratum, it is not surprising that relatively high settlement occurred even at the site with lowest settlement (48%); it is not possible to infer whether the measured soundscape-related effect (i.e., increase from 48% to 63% settlement across treatments) would have a sizable population-scale effect in the wild. How the soundscape might affect the settlement behaviour and attachment of free-swimming larvae that have the flexibility to reject substrate, resuspend, and continue searching, remains to be investigated. Moreover, given that the genus *Orbicella* apparently has high rates of post-settlement mortality (Miller, 2014), the importance of any particular cue to recruitment patterns is even more uncertain based on settlement assays. Future studies that examine the outcomes of habitat selection, as well as the integration and redundancy of multiple cues, will be critical to better interpretation of the ecological significance of settlement rates measured in experiments using cultured larvae.

Identifying the specific soundscape components that drive larval responses, and the scales over which soundscapes can influence settlement patterns, will require further investigation using laboratory and field experiments that evaluate larval responses to manipulations of specific soundscape properties (sound levels and frequencies). Because the full bandwidth sound pressure levels correspond to the sites with the highest and lowest larval settlement, and the spectral composition of these sites also differs, it is unknown if larvae respond to sounds of a particular frequency or to elevated acoustic energy in general. It is not clear from this study whether coral larvae, like fish, crustacean, and molluscan larvae, are able to distinguish the sounds from different habitats, or if the effect reflects a preference for locations with higher acoustic energy. Even so, such a settlement preference could be an adaptive response since healthier, more productive reefs can have higher sound levels (Piercy et al., 2014; Kaplan et al., 2015). Future investigations should evaluate the discriminatory abilities of coral larvae to soundscapes, and the differences found here in acoustic variables and the settlement responses detected between sites provide a framework for generating hypotheses about the habitat-related acoustic characteristics that could influence coral settlement patterns. The acoustic differences between the sites likely relate to differences in abundance of soniferous species, the structure and amount of hard reef substrate with which hydrodynamic forces interact to generate noise, and anthropogenic inputs. Further work is required to isolate specific sound sources, correlate soundscape properties to habitat quality metrics and, in turn, link them to coral settlement patterns. Whether the settlement response is influenced by acoustic cues in the water, the substrate, or both, is an additional open question.

Differences in the amount of larval settlement in chambers corresponded most closely to the relative differences between sites in the lower frequency band SPL (25–1,000 Hz; CW<CE<WF), which suggests that soundscape components in this range are responsible for enhancing larval settlement, rather than higher frequency sound sources. Because the sounds in this range include reef fish calls (Lobel, Kaatz & Rice, 2010), hydrodynamic influences (e.g., current interacting with reef structure), and anthropogenic noise (e.g., vessels, generator noise), there are a variety of possible signals to which coral larvae could be attracted. Alternatively, it is possible that the soundscape at the site with the lowest settlement (CW) had a local sound source that is a deterrent to larval settlement, and this could act to diminish settlement at the site. It is also important to consider that in addition to overall sound levels and frequencies, organisms may respond to other relevant soundscape complexity, such as the timing or pattern of particular sounds. Interestingly, the site with the healthiest reef and highest settlement in this experiment is also adjacent to a desalination plant whose operations likely contribute constant low frequency noise to the reef soundscape, and is a possible source of the 60–70 Hz peak recorded only at this site. Previous reports have shown the low frequency vibrations produced by vessel generators (20–100 Hz) to increase the settlement of a variety of sessile invertebrates to ship hulls (Wilkens, Stanley & Jeffs, 2012; McDonald et al., 2014). Similarly, acoustic input from anthropogenic sources could be contributing to the elevated larval settlement in the chambers placed at the noisier Water Factory site but this cannot be resolved by the current study.

The findings presented here add to the single previous study of the swimming response of coral larvae toward reef sound (Vermeij et al., 2010), and together they implicate acoustic variables in the selection of settlement habitat and, in turn, coral recruitment dynamics. Coral settlement remains poorly understood; however, it is clear that distributional patterns are produced, in part, by active habitat selection and settlement preferences by larvae. While coral larval swimming abilities are insufficient to move against currents, delayed settlement until encounter of habitat cues, such as sound, represents a mechanism by which these weakly-swimming organisms can affect their dispersal and distribution (Kingsford et al., 2002). Because the soundscape can be indicative of the broader habitat characteristics, it is a valuable source of sensory information, providing a signal that supplements the information garnered from other cues (e.g., colour, biofilm, texture) at smaller spatial scales (e.g., centimeters). Acoustic cues might also present information about the hydrodynamic conditions, reef topography and depth variation, predators, and reef community composition.

This study represents an initial step in establishing the importance of soundscape variation to larval settlement for a reef-building coral, and provides the foundation to examine the specific signals and responses underlying the differential responses. That specific components of the soundscape may enhance or impede settlement is a novel avenue of research to gain a better understanding of larval recruitment, as well as the potential adverse effects of anthropogenic noise pollution, and beneficial effects of conserving marine soundscapes. Moreover, studies such as this showing that larvae are selective at settlement and influenced by habitat variables underscore the need to consider the distribution of habitat-related cues in biophysical models used to predict and understand dispersal, population connectivity and recruitment patterns.

##  Supplemental Information

10.7717/peerj.2557/supp-1 Data S1 Coral settlement raw data

## Additional Information and Declarations

### Field Study Permissions

The following information was supplied relating to field study approvals (i.e., approving body and any reference numbers): All field collections and experiments were conducted under the permissions and collecting permit 48584 granted to CARMABI by the Government of Curaçao (Ministry of Health, Environment, and Nature).

### Data Availability

The following information was supplied regarding data availability: The raw data has been supplied as a Supplementary File.
